# Temporal Effects of a *Begomovirus* Infection and Host Plant Resistance on the Preference and Development of an Insect Vector, *Bemisia tabaci*, and Implications for Epidemics

**DOI:** 10.1371/journal.pone.0142114

**Published:** 2015-11-03

**Authors:** Saioa Legarrea, Apurba Barman, Wendy Marchant, Stan Diffie, Rajagopalbabu Srinivasan

**Affiliations:** Department of Entomology, University of Georgia, Tifton, Georgia, United States of America; Institute of Vegetables and Flowers, Chinese Academy of Agricultural Science, CHINA

## Abstract

Persistent plant viruses, by altering phenotypic and physiological traits of their hosts, could modulate the host preference and fitness of hemipteran vectors. A majority of such modulations increase vector preference for virus-infected plants and improve vector fitness, ultimately favouring virus spread. Nevertheless, it remains unclear how these virus-induced modulations on vectors vary temporally, and whether host resistance to the pathogen influences such effects. This study addressed the two questions using a *Begomovirus*-whitefly-tomato model pathosystem. *Tomato yellow leaf curl virus* (TYLCV) -susceptible and TYLCV-resistant tomato genotypes were evaluated by whitefly-mediated transmission assays. Quantitative PCR revealed that virus accumulation decreased after an initial spike in all genotypes. TYLCV accumulation was less in resistant than in susceptible genotypes at 3, 6, and 12 weeks post inoculation (WPI). TYLCV acquisition by whiteflies over time from resistant and susceptible genotypes was also consistent with virus accumulation in the host plant. Furthermore, preference assays indicated that non-viruliferous whiteflies preferred virus-infected plants, whereas viruliferous whiteflies preferred non-infected plants. However, this effect was prominent only with the susceptible genotype at 6 WPI. The development of whiteflies on non-infected susceptible and resistant genotypes was not significantly different. However, developmental time was reduced when a susceptible genotype was infected with TYLCV. Together, these results suggest that vector preference and development could be affected by the timing of infection and by host resistance. These effects could play a crucial role in TYLCV epidemics.

## Introduction

Phytoviruses transmitted persistently often tend to alter the phenotypic and physiological traits of their host plants thus making them more suitable to their arthropod vectors, particularly hemipterans [1–9]. Visual, volatile, and gustatory cues from plants infected by such viruses make them more apparent to their vectors and also affect vector fitness [10–18]. Even though a few studies have documented virus-induced direct effects on vectors [7,19,20], the majority of studies seem to attest that virus-induced effects on vectors are mediated via hosts. Studies that documented host-mediated virus-induced effects on vector preference and/or fitness were, more often than not, conducted using expressive hosts or hosts that display flamboyant symptoms upon virus infection [8–11,13,14,18]. It is unclear if such observed effects on vector preference and/or fitness would also be noticeable in not-so expressive hosts, including host genotypes that exhibit resistance to the pathogen. Further, most experiments that documented persistent virus induced-effects on vectors were conducted in a single time frame post host inoculation. It is also not clear if such induced effects are sustained over time. This study aims to address the two questions using a *Begomovirus*-whitefly-tomato model pathosystem.

*Tomato yellow leaf curl virus* (TYLCV) is a single stranded monopartite DNA virus in the Genus *Begomovirus* and family *Geminiviridae* that causes yellow leaf curl disease in tomato plants. Infected plants are characterized by yellowing, upward leaf curling, and stunting [21–24]. The sweetpotato whitefly, *Bemisia tabaci* (Hemiptera: Aleyrodidae), transmits TYLCV, in a persistent and circulative manner [25]. TYLCV is acquired when the whitefly feeds on the phloem tissue. Upon acquisition, the whitefly remains associated with the virus for several weeks up to its entire lifespan [26]. Replication of viral particles within the insect vector has also been recently acknowledged [27].

A number of studies have examined the effects of *Begomovirus* infection on whiteflies. In several cases plants were deemed to be more attractive to whiteflies, and the vira1infection in plants improved the performance of whiteflies [9, 16, 28–31]. However, in some cases, neutral and negative effects of TYLCV infection on the preference and fitness of whiteflies were also documented [16, 29, 32–34]. On the whole, effects on vector fitness seem to vary with several factors such as the whitefly biotype, the species of *Begomovirus*_,_ and the plant host chosen to evaluate such interactions [35]. For instance, *Begomovirus* infection differentially affected the performance and preference of the whiteflies depending on the biotype of whiteflies [33, 34, 36, 37]. *Tomato yellow leaf curl china virus* (TYLCCNV) infection in tobacco plants was correlated with negative fitness effects on the indigenous whitefly biotype ZHJ1 when compared with the b-biotype in China [33]. Another *Begomovirus*, *Tomato curly shoot virus* (TbCSV), infection in tomato was also correlated with increased fitness effects on the invasive b-biotype whiteflies than the indigenous biotype ZHJ1 [37]. Most of these whitefly-TYLCV studies were conducted at a single time point, often at the time of substantial symptom expression on plants. Whitefly preference to virus-infected plants before and after substantial symptom expression is rather unknown. This study attempts to elucidate the temporal effects of TYLCV infection in susceptible and resistant genotypes on virus accumulation, virus acquisition by whiteflies, whitefly preference, as well as the effects of TYLCV infection on whitefly development.

Most pathosystems associated with studies on vector- virus (persistent)-host interactions typically involve a host plant that is very susceptible to the pathogen. Assuming that effects on the preference and fitness of vectors are aided by phenotypical and physiological alterations of the host, it would be worth investigating whether such effects on vectors are noticeable with hosts that are recalcitrant to infection. This study addresses the issue by using tomato genotypes that exhibit recalcitrance or resistance to TYLCV. A number of TYLCV-resistant genotypes with resistance genes (*Ty-1*, *Ty-2* and *Ty-3*) incorporated from wild species have been developed [38, 39]. Previous research has revealed that the bred genotypes are not immune to the virus, but they exhibit mild symptoms upon infection with TYLCV [38, 40]. Moreover, TYLCV could be acquired from resistant genotypes and inoculated to susceptible genotypes [38, 40]. Therefore, TYLCV resistant genotypes could function as inoculum sources and could influence TYLCV epidemics.

**Fig 1:**
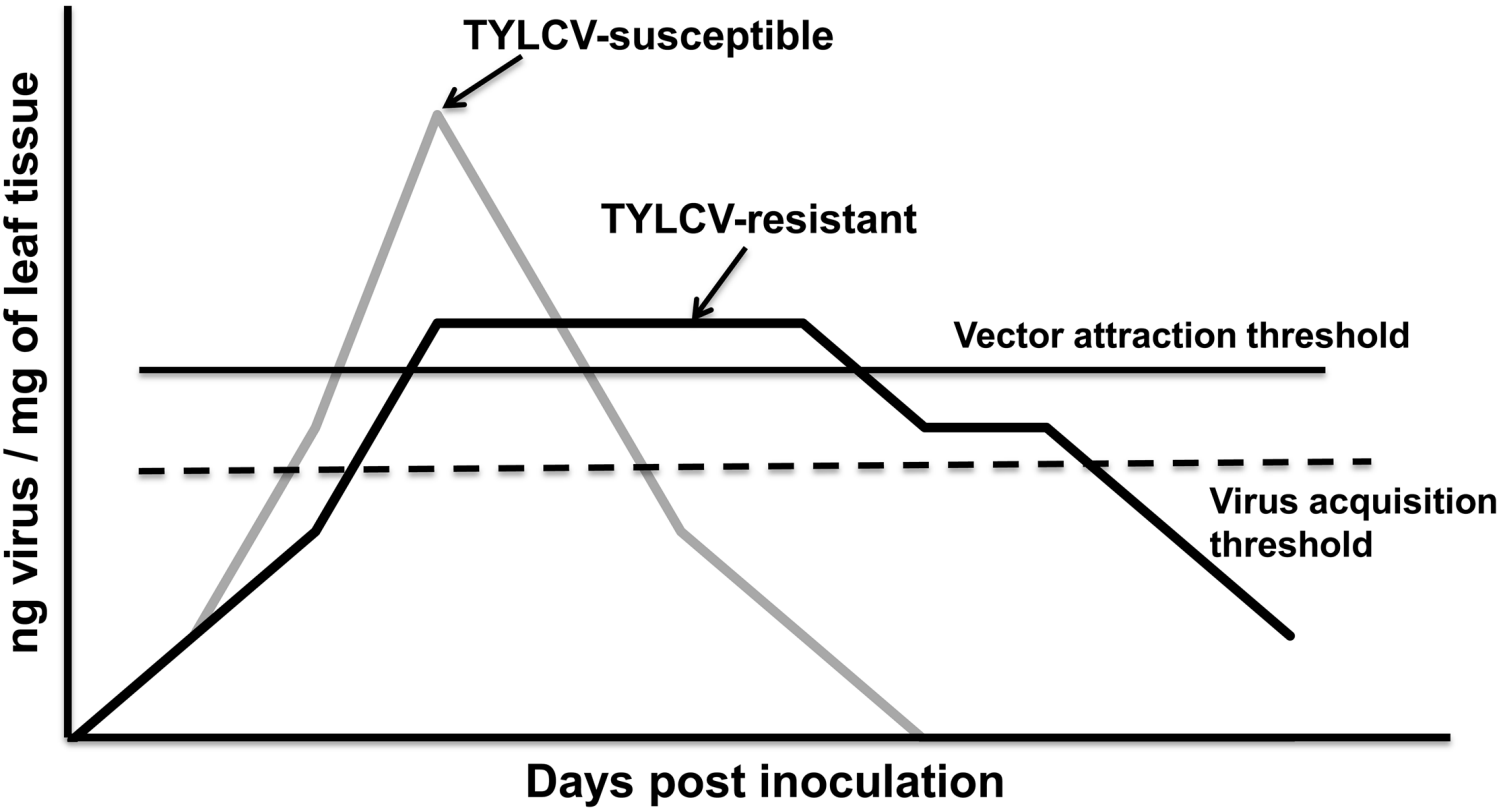
Graphical model of our starting hypothesis. The figure represents accumulation of viral copies in the leaf tissue with time for both, resistant (black line) and susceptible (grey line) genotypes. The threshold for virus acquisition by the vector (dashed black line) and the threshold for vector preference towards infected plants (solid black line) are also shown.

Due to quick disease progression following TYLCV infection and severely altered phenotype in the case of TYLCV-susceptible genotypes than TYLCV-resistant genotypes, and the ability of whiteflies to readily acquire the virus from TYLCV-infected resistant and susceptible genotypes [40], we hypothesized that virus accumulation in TYLCV-resistant genotypes would be above the threshold of TYLCV acquisition by whiteflies for a longer time period than in TYLCV-susceptible genotypes. We also hypothesized that due to less severe symptoms on resistant genotypes; TYLCV-infected resistant genotypes could be phenotypically more attractive to whiteflies for a protracted period than TYLCV-infected susceptible genotypes. Our working hypotheses are illustrated with a graphical model (Fig 1). To test our hypotheses two TYLCV-susceptible and four TYLCV-resistant genotypes were selected. Virus accumulation in all genotypes was estimated at three time intervals i.e. at 3, 6, and 12 weeks post inoculation (WPI), and the ability of whiteflies to acquire TYLCV from both susceptible and resistant genotypes was evaluated. Subsequently, the preference of non-viruliferous and viruliferous whiteflies towards TYLCV-infected and non-infected plants was evaluated at 3, 6, and 12 WPI for both susceptible and resistant genotypes. Developmental time, as a parameter of whitefly fitness, was also measured on TYLCV-resistant and susceptible genotypes.

## Materials and Methods

### Maintenance of plants, virus isolate, and the insect colony

Six commercially available tomato (*Solanum lycopersicum* L.) genotypes were chosen for this study. They included two TYLCV susceptible genotypes, Amelia (Harris Moran Seeds Company, CA, USA) and Florida 47 (Seminis Vegetable Seeds, MO, USA), and four genotypes that are resistant to TYLCV viz. Tygress (Seminis), Security (Harris Moran), Shanty (Hazera Seeds Inc., FL, USA) and Inbar (Hazera). Genes such as *Ty-1* and *Ty-2* confer resistance to TYLCV in resistant genotypes. Plants were grown in Sunshine LP5 Plug Mix (SunGro Horticulture Industries, Bellevue, WA, USA) substrate using 10 cm diameter x 8 cm tall pots and fertilized weekly with water-soluble Miracle-Gro (Scotts Miracle-Gro products, Inc., OH, USA) at 0.5g/l. Plants were maintained in the greenhouse at 25–30°C with a 14h L:10h D photoperiod in whitefly-proof cages 45Lx45Wx90H cm^3^ (Megaview Science Co., Taichung, Taiwan). TYLCV isolate was first collected from a TYLCV-infected tomato plant in a commercial tomato farm in Montezuma (Macon County, GA, USA) in 2009 [34]. TYLCV was maintained since then in a susceptible genotype (Florida 47) through repeated inoculations of 4-leaf stage plants (approximately 4–6 weeks old) with viruliferous whiteflies at 20 whiteflies per plant using clip cages (4 cm tall x 4 cm diameter) [40, 41]. Whiteflies, *Bemisia tabaci* (B biotype), first collected in Tifton, Georgia, were reared on 15 to 20 cm tall cotton plants grown in 10 cm diameter x 8 cm tall pots (Hummert International, Earth City, MO) in whitefly-proof cages under the same conditions described above.

### Virus accumulation in TYLCV-resistant and -susceptible genotypes

Plants used to evaluate TYLCV accumulation were transplanted into 15 cm diameter x 14 cm tall pots to allow adequate plant development throughout the period of study (12 WPI). Plants were inoculated using viruliferous whiteflies as described above. A sample of 100 mg of young leaf tissue in the apex region was collected. Total DNA from samples was immediately extracted using DNeasy plant minikit (Qiagen,Valencia, CA) following the manufacturer guidelines. Six plants were sampled for each cultivar at each time period (3, 6, and 12 WPI), and the experiment was repeated once (n = 12 for each genotype).

Primers for partial TYLCV amplification by real time PCR were designed using Primer-BLAST [42]. Primers flanking a 102 bp region in the C2 ORF were selected (Table 1). Real time PCR was performed using SSoFast™EvaGreen^®^Supermix (Bio-Rad Laboratories, Hercules, CA). Mastermix buffer (2x) was combined with forward and reverse primers (final concentration of 0.5 μM), 1 μl of DNA, and nuclease-free distilled water for a final volume of 20 μl. Quantitative PCR was conducted using Mastercycler^®^ ep realplex^4^ (Eppendorf, Hauppauge, NY). An initial denaturation step (2 min at 98°C) was followed by 40 cycles of denaturation (15 sec at 98°C), and a combined step of annealing and extension at 65°C for 20 sec. Finally, melting curve analysis was conducted to evaluate the specificity of fluorescence signal. Each sample tested was duplicated, and the absolute number of copies present in each sample was quantified. For this purpose, a standard curve was created by including a series of eight 10-fold dilutions of pCR^®^2.1-TOPO^®^ plasmids containing C2 fragments. An approximately 700 bp fragment that included the C2 ORF was amplified using PCR SuperMix (Invitrogen, CA, USA) and the primer pair C2-1159, C2-1853 described in Table 1. To achieve a final volume of 25 μl, 1 μl of each primer (at a final concentration of 0.4μM) were added to 22μl of the reaction buffer (1.1x) with 1 μl of DNA. The reaction was performed in an Eppendorf Mastercycler Pro thermocycler (Eppendorf, Hauppauge, NY) with an initial denaturation at 94°C for 2 min followed by 35 cycles of 94°C for 30 sec, 60°C for 30 sec, and 72°C for 1 min and a final extension step at 72°C for 5 min. The amplified product was ligated to pCR^®^2.1-TOPO^®^ (Invitrogen, Carlsbad, USA) plasmid and transformed to *Escherichia coli* chemically competent cells (TOP10) following manufacturer’s guidelines. Plasmid DNA was extracted using GeneJet Plasmid miniprep kit (Fermentas, MA, USA) and linearized after digestion with Hind III restriction enzyme (Promega, Madison, WI). DNA concentration in the samples was measured in ng/μl using Nanodrop (Thermo Scientific, Wilmington, DE), and the number of copies was estimated based on the formula: number of copies = (amount in ng * 6.022x10^23^)/(length of vector in bp *1x10^9^*650), in which the weight of a base pair (bp) is assumed to be 650 Da [43].

**Table 1:** Primers designed for the amplification of C2 ORF of TYLCV.

Name	Sequence (5’ --- 3’)	nt	Target gene	Product size (nt)
TYLC-C2-For	GCAGTGATGAGTTCCCCTGT	20	C2 ORF	102
TYLC-C2-Rev	CCAATAAGGCGTAAGCGTGT	20	C2 ORF	
C2-1159	CATGATCCACTGCTCTGATTACA	23	C2 ORF	694
C2-1853	TCATTGATGACCTAGCAAAG	20	C2 ORF	

### Whitefly developmental time on TYLCV-resistant and -susceptible tomato genotypes

The time required for *B*. *tabaci* adult development was studied on TYLCV-resistant and -susceptible genotypes. For this purpose, 6-week-old plants maintained in 15 x 14 cm pots in a growth chamber (Percival, Perry, IA) at 26± 0.1°C (mean ± SD) with 14:10 h (L:D) photoperiod were used. Three plants per cultivar were used, and three clip-cages containing two adult females were attached to each plant allowing an oviposition period of 48 hours. Once adult whiteflies and cages were removed, six eggs in each cage were randomly selected and monitored daily until adult emergence.

### TYLCV acquisition by whiteflies from TYLCV-infected resistant and susceptible genotypes

TYLCV acquisition by whiteflies was also evaluated at the three time intervals specified above. Whiteflies were introduced in clip-cages and attached to leaflets on TYLCV infected plants and given an acquisition access period (AAP) of 72 hours. Whiteflies in cages were subsequently transferred to cotton (a non-TYLCV host plant) leaves for another 72 hours. Whitefly DNA was extracted using DNeasy blood and tissue mini kit (Qiagen, CA, USA) pooling five insects in each sample following manufacturer’s instructions. Three samples (15 whiteflies) were processed for each cultivar and for each time interval. The experiment was repeated once (n = 6 for each genotype). Each sample tested was duplicated, and the absolute number of copies in each sample was quantified using qPCR as described for plant tissue samples.

### Whitefly settling

**Fig 2:**
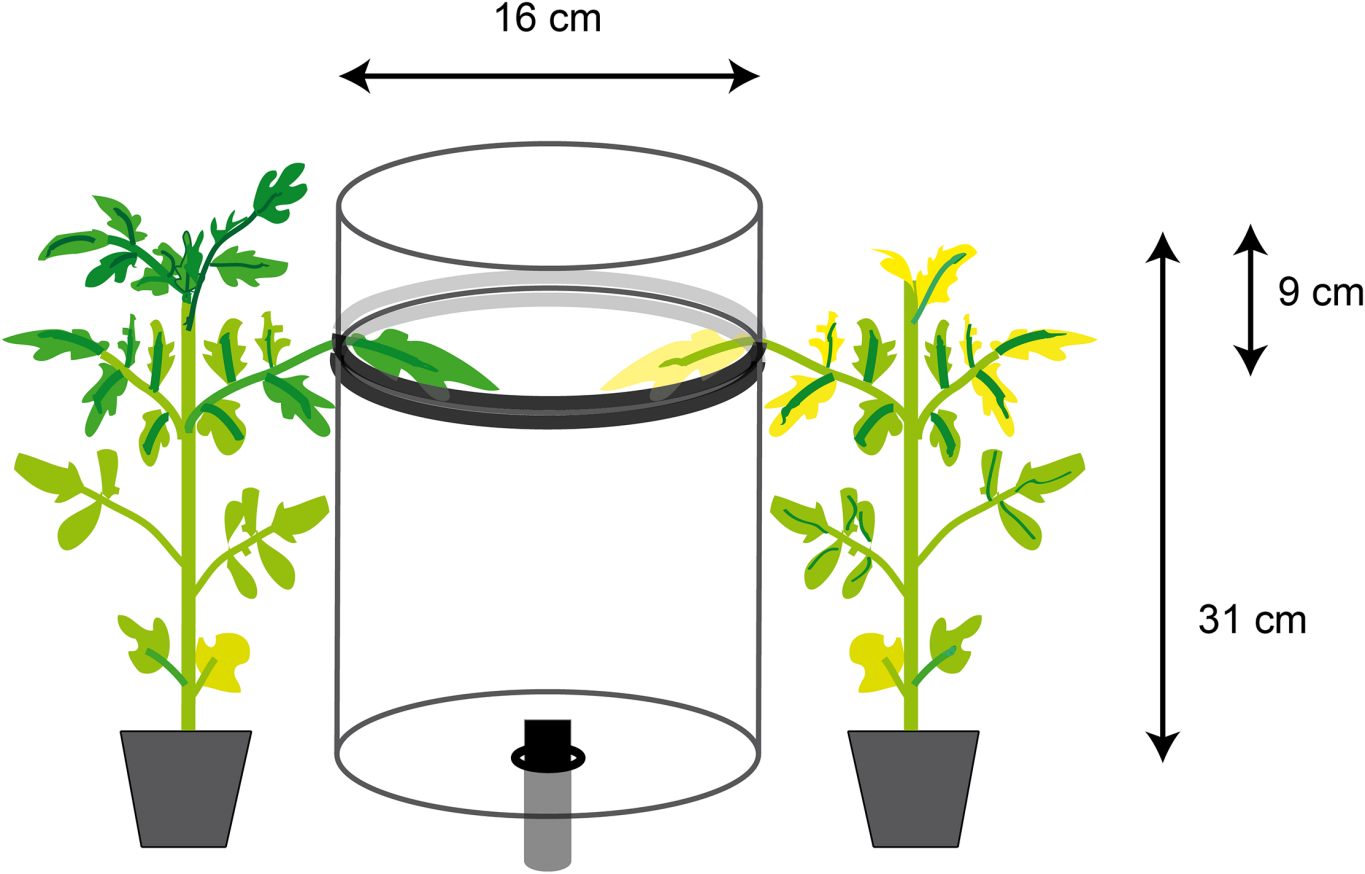
Schematic representation of the whitefly settling assay setup. The clear plastic (Mylar^®^film) cylinder (16 cm diameter and 31 cm tall) was glued to petri dish plates (16 cm diameter) in both ends creating an enclosed structure that was sectioned at a distance 9 cm from the top and surrounded by foam at sectioned ends. Two tomato leaves (infected and non-infected) attached to respective plants were placed across each other. Whiteflies were released in a glass vial placed through an opening at the bottom centre of the structure.

Whitefly settling assays were conducted at three time intervals: 3, 6 and 12 WPI. Two tomato genotypes were used viz. a TYLCV susceptible (Florida 47) and a TYLCV resistant genotype (Security). Settling assays were carried out with either viruliferous or non-viruliferous whiteflies. In the former case, whiteflies were given an AAP of 72h on symptomatic TYLCV-infected tomato plants. Non-viruliferous whiteflies were maintained on cotton as described previously. TYLCV infection or lack thereof in a subset of whitefly samples was confirmed by PCR (using C2-1159, C2-1853 primers) once the experiment was completed. The settling arena included a plastic cylinder 16 cm diameter x 31 cm tall. The cylinder was sectioned at 9 cm from the top and a strip of rubber foam (1.9 mm wide x 11.1 mm thick) was placed surrounding the edges to provide stability. This allowed to position leaflets from each genotype while being attached to the plant. The leaflets were positioned opposite to each other (Fig 2). One hundred whiteflies were collected into a 10 ml glass vial (VWR, Radnor, PA) and sealed with paraffin film (Parafilm M^®^, American Can Company, Greenwich, CT). After cooling the vial on ice for a few minutes, whiteflies were allowed to enter the arena from the vial placed at the bottom. The number of whiteflies settling on each plant was recorded after 24 hours. Five replications were performed for each vector type (viruliferous or non-viruliferous) and repeated twice (n = 15) for each time interval (3, 6 and 12 WPI). Experiments were conducted under laboratory conditions at 20–25°C and a photoperiod of 12h L:12 h D.

### Effect of TYLCV on whitefly developmental time

Effects of TYLCV infection on whitefly development were studied on a resistant genotype (Security) in comparison with a susceptible genotype (Florida 47). Three plants from each genotype with and without TYLCV infection were used. Infected plants were obtained by whitefly-mediated inoculations as described earlier. The experiment was initiated six WPI by attaching three clip-cages on each plant with two females in each cage for 48 h. Eggs and immatures were monitored daily following the same methodology as explained above. The experiment was performed in a growth chamber at (26 ± 3°C) (mean ± SD) and a photoperiod of 14:10 h (L:D).

### Statistical analysis

In general, data from experimental repeats were pooled for each study prior to conducting statistical analysis. Differences in TYLCV copy numbers in various genotypes at each time interval were assessed by subjecting the data to generalized linear mixed model using PROC GLIMMIX with suitable transformation in SAS Enterprise 4.2 (SAS Institute, Cary, USA). Genotypes (treatments) were considered as fixed effects, and replications were considered as random effects. Treatment means were separated using the Tukey Kramer option in SAS at α = 0.05. Whitefly developmental time (egg to adult) data for each life stage were subjected to median one-way analysis using PROC NONPAR1WAY in SAS. Genotypes were considered as fixed effects and replicates as random effects. Whitefly settling differences between infected and non-infected leaflets in each genotype at each time interval were evaluated by logistic regression using PROC GENMOD in SAS assuming a binomial distribution and a logit link function. Whitefly developmental time on infected and non-infected plants of each genotype was evaluated using median one-way analysis as described earlier.

## Results

### Virus accumulation in TYLCV-resistant and -susceptible genotypes

**Fig 3:**
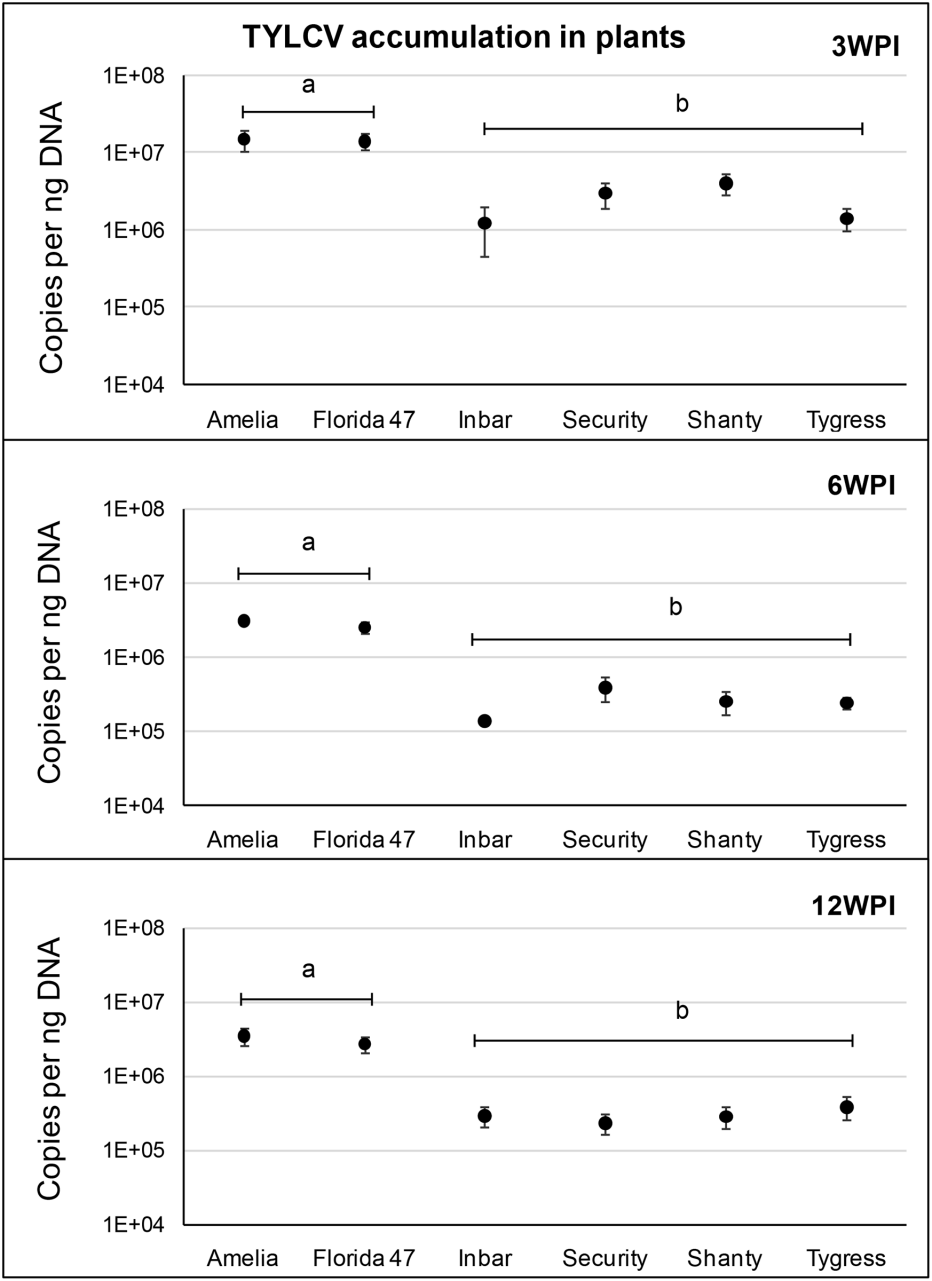
TYLCV accumulation in leaf tissue at 3, 6 and 12 weeks post inoculation (WPI). Circles with standard errors represent average number of TYLCV C2 copies accumulated in leaf samples from TYLCV-resistant and -susceptible genotypes (Amelia and Florida 47). Copy numbers were estimated by qPCR followed by absolute quantification using plasmids containing TYLCV C2 gene inserts as standards. Different letters on bars indicate significant differences in copy numbers among treatments as deduced by Tukey-Kramer grouping (α = 0.05). Y-axis is shown in a logarithmic scale.

Significant differences in virus accumulation were observed among tomato genotypes at 3 weeks (*F* = 11.88; df = 5, 64; *P*<0.0001), 6 weeks (*F* = 43.26; df = 5, 64; *P*<0.0001), and 12 weeks (*F* = 12.71; df = 5, 63; *P*<0.0001) post inoculation, respectively (Fig 3). Susceptible genotypes (Amelia and Florida 47) accumulated greater numbers of viral copies when compared with resistant (Inbar, Security, Shanty and Tygress) genotypes regardless of the time post inoculation. Viral copy numbers did not differ significantly among TYLCV-resistant genotypes (Fig 3). The numbers of viral copies in TYLCV-susceptible genotypes were 3 to 22 times greater than in TYLCV-resistant genotypes. Regardless of the genotype, the copy numbers were greater at 3 weeks post inoculation than at 6 and 12 weeks post inoculation. There was a substantial decrease in viral copy numbers from 3 to 6 weeks, but that was not evident from 6 weeks to 12 weeks (Fig 3).

### Whitefly developmental time on TYLCV-resistant and -susceptible tomato genotypes

*B*. *tabaci* successfully developed from egg to adult on all genotypes. Median developmental time varied with genotypes (Table 2). The total developmental time on Tygress was longer than on other genotypes (Table 2). Nevertheless, developmental time does not seem to be associated with TYLCV resistance, as whitefly median developmental time on three other TYLCV-resistant genotypes (Inbar, Shanty and Security) was very similar to that of susceptible genotypes (Amelia and FL-47) (Table 2).

**Table 2:** Developmental time of whiteflies on different tomato genotypes.

Genotype^W^	N^X^	Egg-adult^Y^	Sum of scores^Z^	Expected under H_0_ ^Z^	Std Dev under H_0_ ^Z^	Mean Score^Z^
Amelia	42	22 (20–30)	25.09	21	515.54	0.60
Florida 47	46	21(19–30)	23.23	23	535.33	0.50
Inbar	50	20 (18–27)	13.20	25	553.71	0.26
Security	54	20 (18–25)	20.20	27	570.81	0.37
Shanty	54	21(19–25)	18.75	27	570.81	0.34
Tygress	54	23.5 (19–33)	49.51	27	570.81	0.91
**Test statistics**						
*Χ* ^*2*^ = 66.65						
Df = 5, 294						
*P*> *Χ* ^*2*^ <0.0001						

^W^Whitefly development was monitored on two TYLCV-susceptible (Amelia and Florida 47) and four TYLCV-resistant tomato genotypes.

^X^Number of individual eggs monitored to adulthood on each genotype.

^Y^Median whitefly development time from egg to adult on each genotype with range in parentheses.

^Z^Sum of scores for median one-way analysis, sum of scores expected under null hypothesis that developmental time in all genotypes is not different, standard deviation from null hypothesis and mean scores.

### TYLCV acquisition by whiteflies from TYLCV-infected resistant and susceptible genotypes

**Fig 4:**
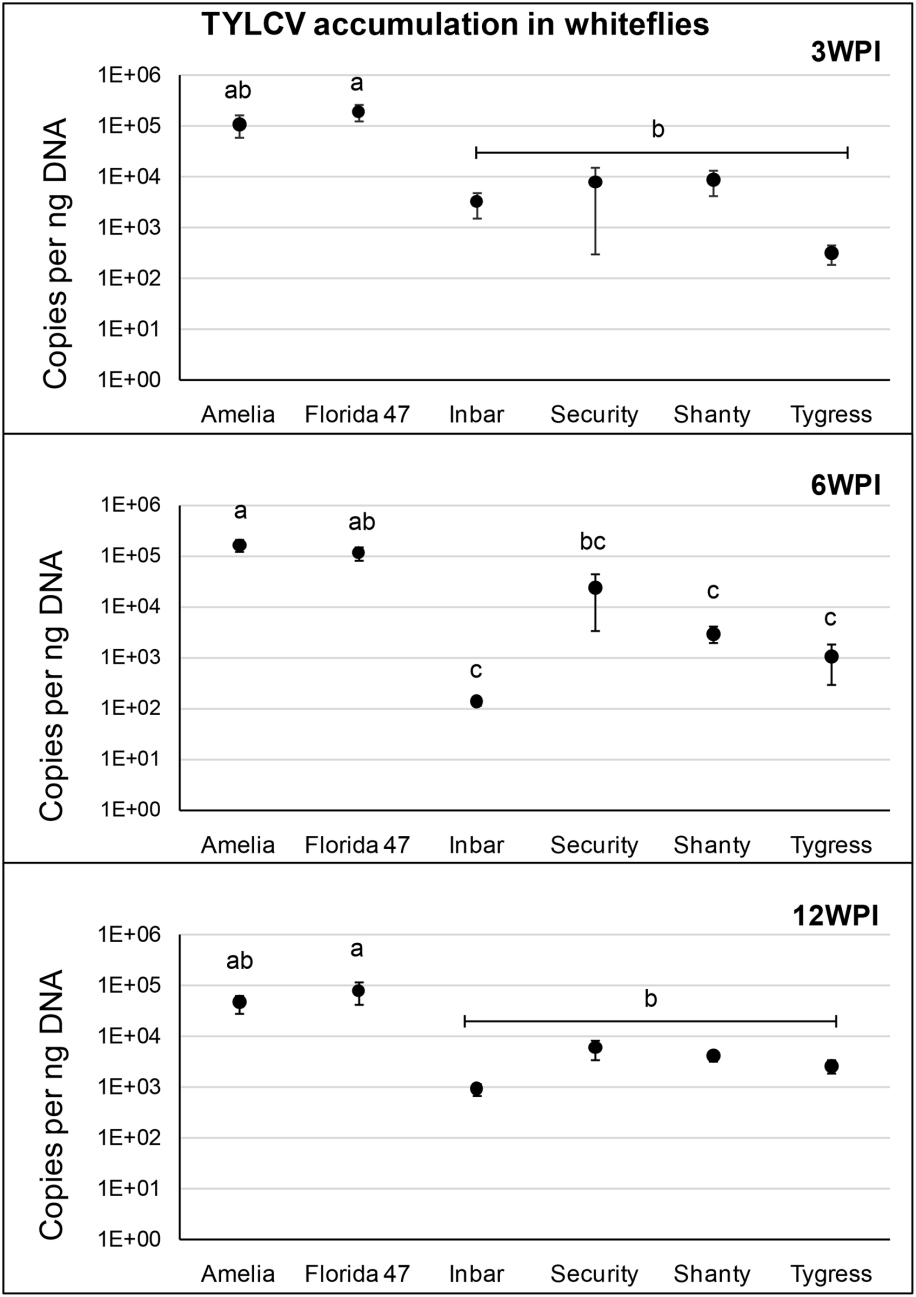
TYLCV acquisition by whiteflies (*B*. *tabaci*) at 3, 6 and 12 weeks post inoculation (WPI). Circles with standard errors represent average number of TYLCV C2 copies accumulated in whitefly samples (5 whiteflies in each sample) that were exposed to a 72 h AAP on TYLCV infected foliage of TYLCV-resistant and -susceptible tomato genotypes followed by 72 h of caging on cotton plants. Copy numbers were estimated by qPCR followed by absolute quantification using plasmids containing TYLCV C2 gene inserts as standards. Different letters on bars indicate significant differences in copy numbers among treatments as deduced by Tukey-Kramer grouping (α = 0.05). Y-axis is shown in a logarithmic scale.

Whiteflies were able to acquire the virus from infected plants regardless of the genotype for all time periods (Fig 4). In general, whiteflies acquired more TYLCV copies from TYLCV-susceptible genotypes than from TYLCV-resistant genotypes at 3 (*F* = 5.31; df = 5, 27; *P* = 0.0020), 6 (*F* = 10.43; df = 5, 30, *P*<0.0001), and 12 (*F* = 4.11; df = 5, 28; *P* = 0.0070) WPI, respectively. Accumulation of TYLCV in whiteflies following a 72h AAP on TYLCV-susceptible genotypes was up to three orders of magnitude greater than in whiteflies that were given an AAP on TYLCV-resistant genotypes. However, the ability of whiteflies to acquire the virus from infected plants of susceptible or resistant genotypes was not influenced by the time of inoculation (Fig 4).

### Whitefly settling

Settling differences between infected and non-infected leaves were evaluated using one TYLCV-susceptible (Florida 47) and one TYLCV-resistant (Security) genotype. In general, TYLCV infection status of whiteflies, TYLCV susceptibility status of resistant and susceptible genotypes, as well as timing post inoculation influenced whitefly settling.

**Fig 5:**
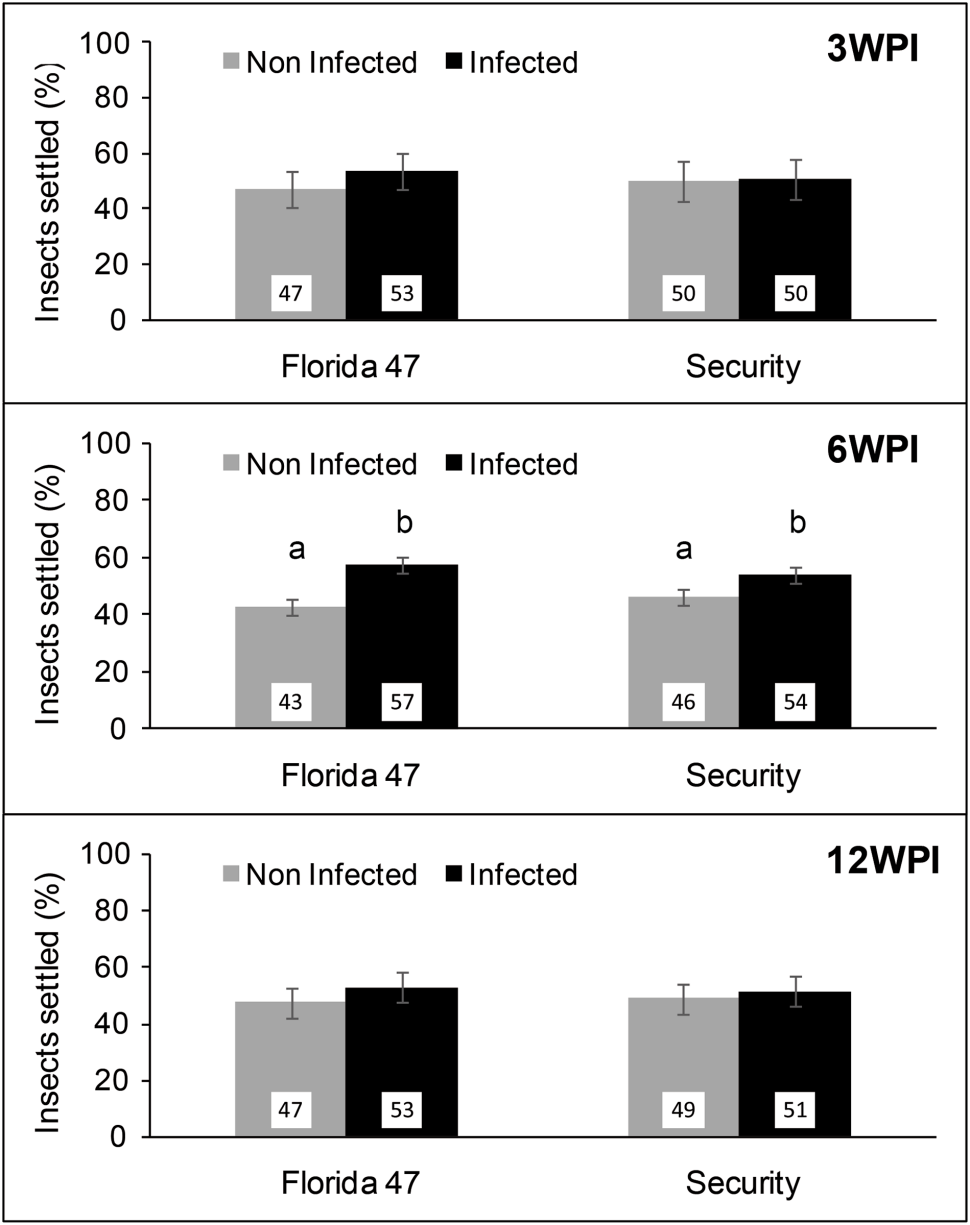
Settling of non-viruliferous whiteflies (*B*. *tabaci*). Bars with standard errors indicate percent settling of non-viruliferous adult whiteflies on TYLCV-infected (black bars) or non-infected (grey bars) leaves of a TYLCV-resistant (Security) and a susceptible (Florida 47) genotype 24h post release. Panels show whitefly settling on infected and non-infected plants after 3, 6, and 12 weeks post inoculation, respectively. Different letters indicate significant differences between treatments at α = 0.05 based on least square means.

No differences in non-viruliferous whiteflies’ settling on TYLCV-infected or non-infected Florida 47 (*Χ* ^2^ = 0.87; df = 1,28; *P =* 0.3516) and Security (*Χ* ^2^ = 0.04; df = 1,28; *P =* 0.8496) leaves were observed at 3 weeks post inoculation (Fig 5). However, at 6 weeks post inoculation, non-viruliferous whiteflies preferentially settled on TYLCV-infected leaves than on non-infected leaves of Florida 47 (*Χ* ^2^ = 60.27; df = 1,28; *P<*0.0001) and Security (*Χ* ^2^ = 25.12; df = 1,28; *P<*0.0001), respectively (Fig 5). At 12 weeks post inoculation, no preferential settling was observed on Florida 47 (*Χ* ^2^ = 0.61; df = 1, 26; *P =* 0.4412) and Security (*Χ* ^2^ = 0.34; df = 1,28; *P =* 0.5614) (Fig 5).

**Fig 6:**
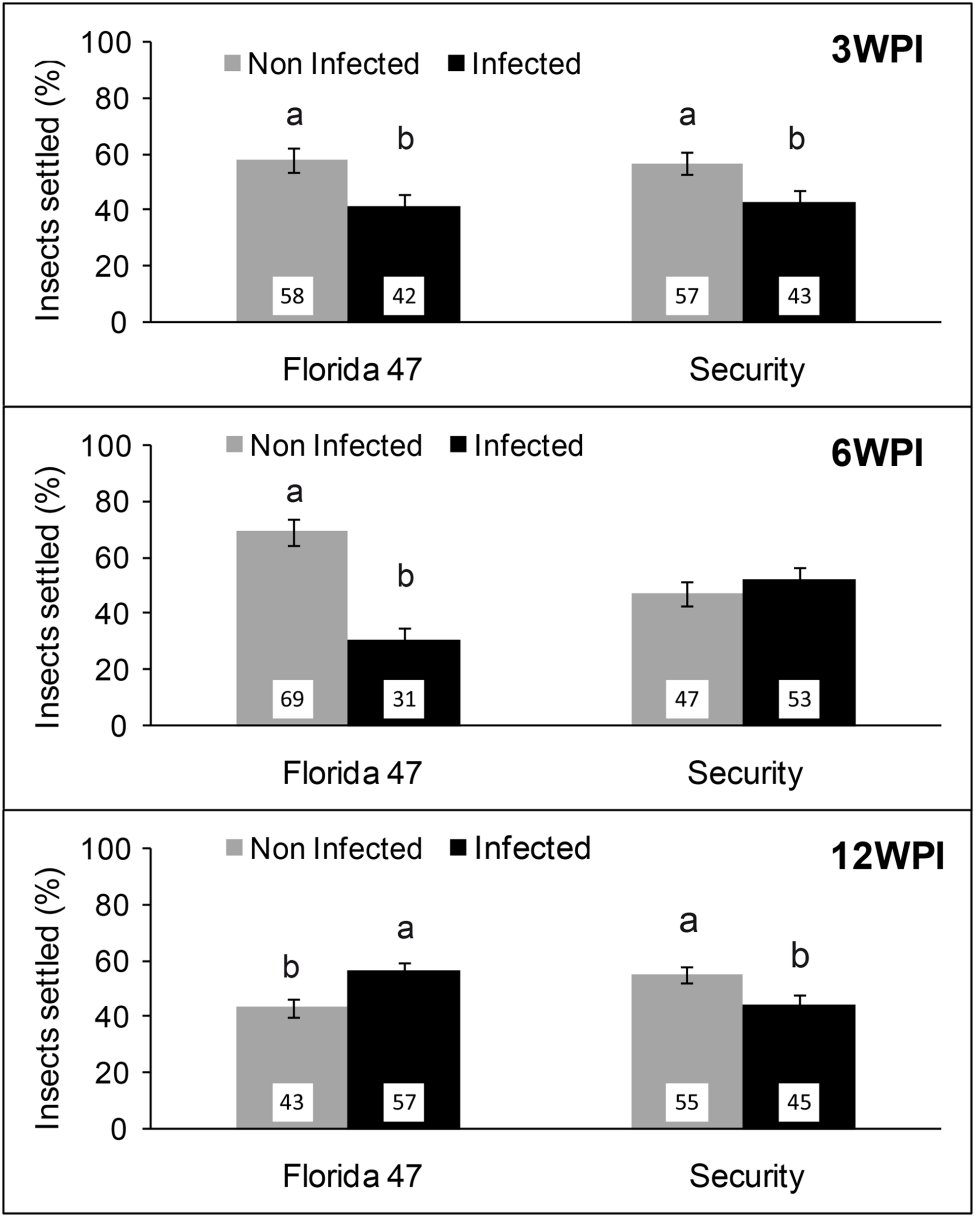
Settling of viruliferous whiteflies (*B*. *tabaci*). Bars with standard errors indicate percent settling of viruliferous whiteflies on TYLCV-infected (black bars) or non-infected (grey bars) leaves of a TYLCV-resistant (Security) and a susceptible (Florida 47) genotype 24h post release. Panels show whitefly settling on infected and non-infected plants after 3, 6, and 12 weeks post inoculation, respectively. Different letters indicate significant differences between treatments at α = 0.05 based on least square means.

More viruliferous whiteflies settled on non-infected leaves, a converse effect than what we observed with non-viruliferous whiteflies, which preferred to settle on TYLCV symptomatic leaves (Fig 6). At three weeks post inoculation, viruliferous whiteflies preferentially settled on non-infected leaves versus TYLCV-infected leaves of both the susceptible (*Χ* ^2^ = 65.94; df = 1,28; *P<*0.0001) and the resistant genotype (*Χ* ^2^ = 58.60; df = 1,28; *P<*0.0001), respectively. Similarly, at 6 weeks post inoculation, viruliferous whiteflies preferentially settled on non-infected leaves when compared with TYLCV-infected leaves of Florida 47 (*Χ* ^2^ = 255.57; df = 1,28; *P<*0.0001). However, this difference was not significant on Security (*Χ* ^2^ = 2.94; df = 1,28; *P =* 0.0867). At 12 weeks post inoculation, settling preference of viruliferous whiteflies switched towards infected leaves in the case of Florida 47 (*Χ* ^2^ = 26.62; df = 1,28; *P<*0.0001). In contrast, in the case of the resistant genotype, Security, more whitefly settling was observed on non-infected leaves than on TYLCV-infected leaves (*Χ* ^2^ = 17.51; df = 1,13; *P<*0.0001).

### Effect of TYLCV on whitefly developmental time

Whitefly development was monitored on infected and non-infected leaves of one TYLCV-susceptible (Florida 47) and one TYLCV-resistant (Security) genotype. Whitefly median developmental time varied with TYLCV infection, but not in both genotypes. The total developmental time from egg to adulthood was less on TYLCV-infected Florida 47 leaves than on non-infected leaves (*Χ* ^2^ = 6.50; df = 1,81; *P* = 0.0109) (Table 3). No difference in whitefly developmental time from egg to adult was observed when reared on TYLCV-infected and non-infected leaves of the TYLCV-resistant genotype, Security (*Χ* ^2^ = 0.81; df = 1,60; *P* = 0.3671) (Table 3).

**Table 3:** Developmental time of whiteflies on TYLCV-resistant and -susceptible genotypes with and without TYLCV infection.

Genotype^V^	TYLCV infection^W^	N^X^	Egg-adult^Y^	Sum of scores^Z^	Expected under H_0_ ^Z^	Std Dev under H_0_ ^Z^	Mean Score^Z^
Florida 47	Yes	44	23 (20–29)	13.57	19.26	2.23	0.37
Florida 47	No	39	25 (19–34)	27.42	21.73	2.23	0.62
*Χ* ^*2*^	6.48						
Df	1, 82						
*P*> *Χ* ^*2*^	0.0109						
Security	Yes	32	29 (21–38)	16.50	15.24	1.94	0.55
Security	No	30	26 (20–35)	13.50	14.75	1.94	0.43
*Χ* ^*2*^	0.8135						
Df	1, 60						
*P*> *Χ* ^*2*^	0.3671						

^V^Whitefly development was monitored on TYLCV-susceptible (Florida 47) and TYLCV-resistant tomato (Security) genotypes.

^W^Whitefly development was monitored on two genotypes (TYLCV-susceptible and resistant) with and without TYLCV infection.

^X^Number of individual eggs monitored to adulthood on each genotype.

^Y^Median whitefly development time from egg to adult on each genotype with range in parentheses.

^Z^Sum of scores for median one-way analysis, sum of scores expected under null hypothesis that developmental time in genotypes with and without TYLCV infection is not different, standard deviation from null hypothesis and mean scores.

## Discussion

**Fig 7:**
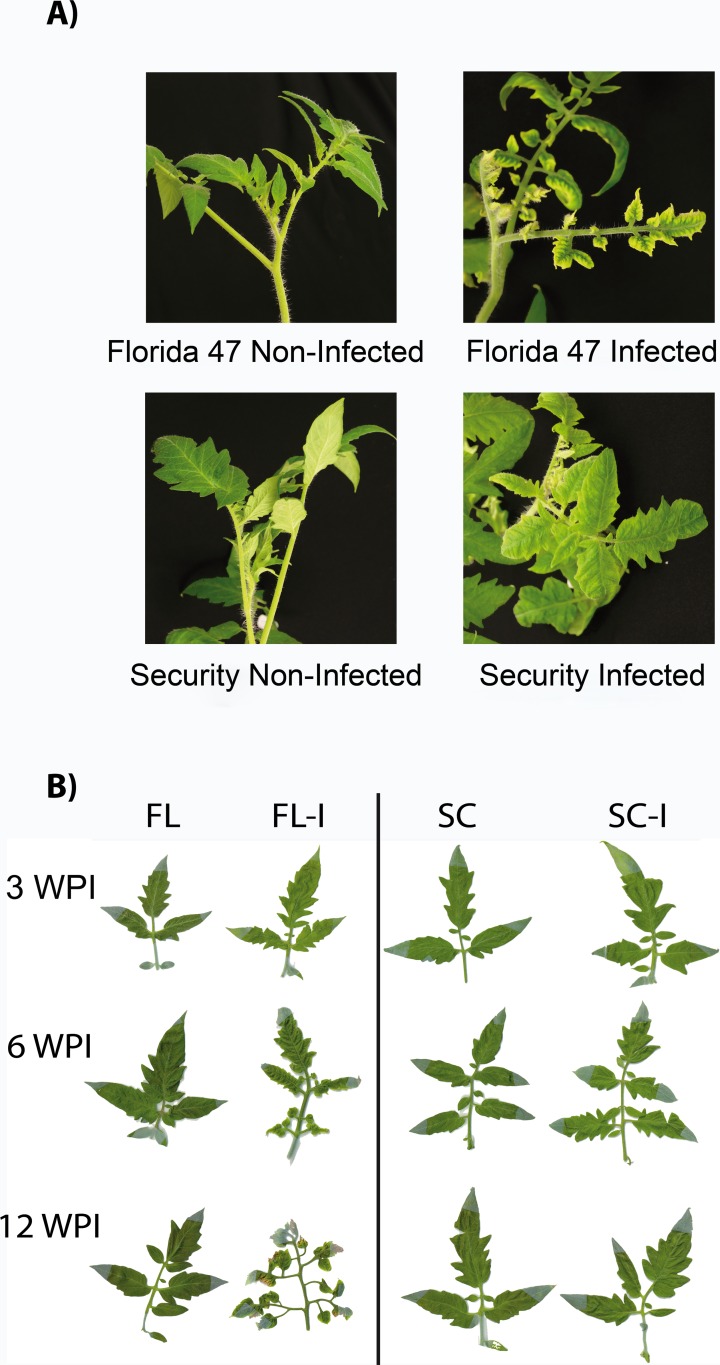
Photos of TYLCV-symptomatic and non-symptomatic leaves used in experiments. (A) TYLCV associated symptoms on infected plants six weeks post inoculation in a susceptible (Florida 47) and a resistant (Security) genotype, as compared with non-infected plants. (B) Representative example of leaflets used in settling assays at 3, 6, and 12 weeks post inoculation (WPI). A TYLCV-susceptible (Florida 47, FL) and a resistant (Security, SC) genotype were chosen for the study.

The main objectives of this study were to examine the temporal effects of a persistent virus (TYLCV) infection as well as the effects of host (tomato) susceptibility to the virus on vector (whitefly) preference and performance. Previous research has shown that semi-dominant genes such as *Ty-1* confer resistance to TYLCV by limiting symptom expression and restricting virus accumulation [38, 40, 44]. However, the temporal impact on virus accumulation and the subsequent effects on vector preference and performance were not investigated. In this study, TYLCV copy numbers at three representative time points in two TYLCV-susceptible and four TYLCV-resistant genotypes were estimated using quantitative PCR. Results indicated that, irrespective of virus-susceptibility status of the host plant, viral copy numbers dropped after the initial observation at three weeks post inoculation. Nevertheless, most severe symptoms appeared beyond three WPI (~from 5 to 6 weeks) (Fig 7). At all time intervals tested, TYLCV copy numbers were less in TYLCV-resistant genotypes than in TYLCV-susceptible genotypes.

This study also assessed whether tomato genotypes harbouring resistance genes to TYLCV itself had any impact on whitefly development by measuring the developmental time. Whiteflies on non-infected resistant genotypes developed both faster (i.e Security) and slower (i.e. Tygress) than on the susceptible genotype (Amelia). These findings corroborate previously observed results that resistant genotypes by themselves did not affect whitefly development [38]. Another previous study also observed no relationship between *B*. *tabaci* natural infestation and TYLCV resistance in a field experiment [40]. Therefore, the obtained results reiterate that the presence of TYLCV resistance genes is not a limiting factor for the development of its insect vector.

Even though the development of whiteflies was not influenced by the presence or absence of the resistant gene, TYLCV acquisition ability of whiteflies seems to be dependent upon the susceptibility status of tomato genotypes. Results from this study demonstrated that whiteflies acquired fewer viral copies from TYLCV-infected resistant genotypes than from susceptible genotypes irrespective of the time post inoculation. This suggested that TYLCV acquisition is a function of virus accumulation on the plant tissue they feed on. Kollenberg et al. recently proved this hypothesis by exposing whiteflies to feed from a solution of purified virions at a specific concentration and thereafter quantifying virus load in the insects [45]. In addition, previous findings also revealed that whiteflies were able to acquire TYLCV from infected resistant genotypes and inoculate the same to non-infected hosts [40]. Together, these results suggest that virus accumulation in TYLCV-resistant genotypes, though lower than TYLCV-susceptible genotypes, is still above the threshold that would limit virus acquisition by whiteflies.

Given that TYLCV-resistant genotypes could also potentially serve as inoculum sources of the virus, their impact on viral epidemics would further depend on how they influence vector preference and performance. Results from the current study indicated that host susceptibility to the virus differentially affected vector preference, the effects varied temporally and with the infection status of whiteflies. With a TYLCV susceptible genotype, non-viruliferous whiteflies showed a significant preference for infected plants six weeks after inoculation. On the contrary, viruliferous whiteflies significantly preferred non-infected plants at three and six weeks after inoculation. Non-viruliferous vector preference towards virus-infected plants and viruliferous vector preference for non-infected hosts could, in general, favor viral epidemics [19, 46]. The cues that influence such interactions are not known in this system, but a combination of visual, volatile, and/or gustatory (nutrients) cues, as in other persistent virus pathosystems [11,16,18,20], could be influencing whitefly preference as well. Availability of nutrients and defensive compounds are also altered by begomoviruses so that infected plants are more suitable for sustained whitefly feeding [35]. Perhaps, such an alteration could have also influenced the increased settling of non-infected whiteflies on TYLCV-infected tomato plants in susceptible cultivars. In addition to TYLCV infection in plants, TYLCV infection in whiteflies also induced a behavioural change, as infected whiteflies preferentially settled on non-infected plants as early as 3 weeks post infection. Direct changes in behavior (i.e. feeding and locomotory activities) following begomovirus infection have already been described for the b and q biotypes of *B*. *tabaci* [7,28]. Another study demonstrated that viruliferous whiteflies were attracted towards green light [47]; such an attraction could explain why viruliferous whiteflies preferentially settled on non-infected plants in this study. Such behavioural patterns modulated by the virus directly through whitefly infection or indirectly through the host plant, particularly in the early stages of the host, could have implications for viral epidemics.

In contrast to whitefly responses in three and six weeks, TYLCV-induced modulations on whiteflies settling faded away 12 weeks after inoculation. At this point of time, additional factors aside from virus accumulation and symptom expression could have played a role in vector preference and/or settling. For example, whiteflies are known to preferentially feed on plants that are enriched with high concentrations of nitrogen [48,49]. In general, as plants age, their quality for herbivory (including nitrogen availability) decreases [50,51]. In tomato, lower N availability has been correlated with an increase in certain secondary metabolites (i.e. phenolics) [52,53]. In our system, physiological and phenotypical changes that occurred at 12 weeks may have negated preferential settling of non-viruliferous whiteflies to virus-infected plants. Whether the settling patterns observed over time were related to changes in availability of nutrients and/or secondary metabolites needs to be further investigated.

In this study, the preference of viruliferous whiteflies was reversed 12 weeks after inoculation, a slightly higher proportion of insects settled on infected plants of the susceptible genotype. However, the opposite result was found for the recalcitrant cultivar. As explained before, other factors aside from the sole impact of virus infection could have influenced this outcome. Overall, virus-induced effects on whitefly preference were not so robust in the case of the TYLCV-resistant genotype as in the susceptible genotype. The results observed in this study have reshaped our original hypotheses that TYLCV-resistant genotypes would accumulate virus above the threshold of acquisition by whiteflies for a longer period and remain attractive to vectors longer than susceptible genotypes. Our results indicate that both TYLCV-susceptible and TYLCV-resistant genotypes, upon infection, could accumulate virus loads well above the threshold of virus acquisition; however, severity and timing of symptom development in the TYLCV-susceptible genotype could facilitate viral epidemics better than TYLCV-resistant genotypes by manipulating vector preference.

In addition to preference, performance of vectors on TYLCV-resistant and -susceptible genotypes could play a critical role in virus spread. Previous studies indicated direct effect of TYLCV infection in the b-biotype, where a number of whitefly life table parameters were negatively impacted [34] thereby suggesting a pathogenic role of the virus on this biotype. In contrast, TYLCCNV greatly increased the performance by indirectly affecting the plant physiology and by promoting sustained ingestion from the phloem sieve elements [28]. Results from the current study revealed that whiteflies completed their development sooner on virus-infected plants when compared with non-infected plants suggesting a positive fitness effect, presumably by the indirect effect of virus infection mediated through the plant. A number of studies so far have also documented similar positive fitness effects of whiteflies when reared on *Begomovirus*-infected plants than on non-infected plants [7,9,29–31]. A number of them have also reported neutral to negative effects on whiteflies [16,32–34]. A majority of these studies were conducted with susceptible host plants and not with recalcitrant or resistant host plants. When the developmental time of whiteflies was examined on TYLCV-infected and non-infected leaves, no differences in developmental time between infected or non-infected plants were observed in the tomato resistant genotype. These results reiterate that host plant susceptibility to the pathogen could be a major driving factor that influences vector-pathogen interactions.

While it is true that a few studies have documented direct effects of the virus on hemipteran vectors [7,19], much of the evidence available today on vector-pathogen interactions seem to indicate that effects of vectors are facilitated by virus-induced host plant phenotypic or physiological alterations. This study clearly documents that pathogen-induced host effects on vectors preference and performance are not fixed but could rather vary temporally. Also, such interactions could fluctuate tremendously with varying degrees of susceptibility of the host plant to the pathogen. These results point to the limitations of generalizing vector-pathogen interactions by conducting experiments at a single time point as well as using a highly susceptible host. Furthermore, these results contribute to a better understanding of relevant factors (i.e. plant genotype, time post inoculation and infection status of vectors) that may drive epidemics of whitefly-transmitted TYLCV.

## Supporting Information

S1 File Supplementary Excel file with raw data. (XLS)
